# CaMKIIβ in Neuronal Development and Plasticity: An Emerging Candidate in Brain Diseases

**DOI:** 10.3390/ijms21197272

**Published:** 2020-10-01

**Authors:** Olivier Nicole, Emilie Pacary

**Affiliations:** 1 CNRS, UMR5293 Institut des Maladies Neurodégénératives, University of Bordeaux, F-33000 Bordeaux, France; olivier.nicole@u-bordeaux.fr; 2 INSERM, Neurocentre Magendie, U1215, University of Bordeaux, F-33000 Bordeaux, France

**Keywords:** CaMKII, brain, neuronal development, neuronal plasticity, neurodevelopmental disorders, psychiatric diseases

## Abstract

The calcium/calmodulin-dependent protein kinase II (CaMKII) is a ubiquitous and central player in Ca^2+^ signaling that is best known for its functions in the brain. In particular, the α isoform of CaMKII has been the subject of intense research and it has been established as a central regulator of neuronal plasticity. In contrast, little attention has been paid to CaMKIIβ, the other predominant brain isoform that interacts directly with the actin cytoskeleton, and the functions of CaMKIIβ in this organ remain largely unexplored. However, recently, the perturbation of CaMKIIβ expression has been associated with multiple neuropsychiatric and neurodevelopmental diseases, highlighting *CAMK2B* as a gene of interest. Herein, after highlighting the main structural and expression differences between the α and β isoforms, we will review the specific functions of CaMKIIβ, as described so far, in neuronal development and plasticity, as well as its potential implication in brain diseases.

## 1. Features of CaMKIIβ

### 1.1. CaMKIIβ Structure and Properties

The calcium/calmodulin-dependent protein kinase II (CaMKII), which is a serine/threonine protein kinase, is one of the most abundant proteins in the brain [1]. There are four isoforms of CaMKII (α, β, γ, δ) that are encoded by four distinct but highly related genes (*CAMK2A*, *CAMK2B*, *CAMK2G*, *CAMK2D*) located on different chromosomes. Although these isoforms show strong sequence similarities [2], they present different biochemical properties and localization [3,4]. As an example, CaMKIIα is absent from amphibians (*Xenopus laevis*) and it has the most restricted tissue specificity in mammals [2,3].

**Figure 1:**
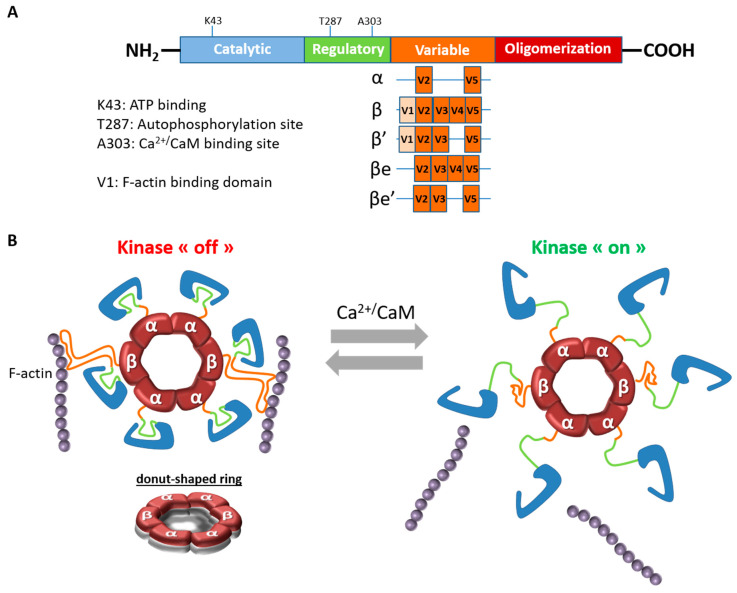
Structural organization of CaMKIIβ. (**A**). Schematic representation of CaMKIIβ structure and its variants. CaMKIIα and CaMKIIβ differ mostly in the variable region. The variable region of CaMKIIα only contains V2 and V5 domains. The variable region V1, which is absent in CaMKIIβe and CaMKIIβ’e, is necessary for the binding to the actin cytoskeleton. (**B**) Calcium/calmodulin-dependent protein kinase II (CaMKII) is organized into large oligomers typically of 12 or 14 subunits. Central to the organization of CaMKII is the hub domain, also known as the association or oligomerization domain, which forms a donut-shaped ring that is the core of the holoenzyme (see side view). The kinase domains are tethered to the central hub by the regulatory segments. In basal condition, the CaMKII holoenzymes, via CaMKIIβ, bind to actin filaments, particularly in dendritic spines. Upon Ca^2+^ influx, Ca^2+^/CaM binds to a CaM-binding element in the regulatory segment of CaMKII, releasing it from the kinase domain and thereby activating the enzyme. At the same time, CaMKII holoenzymes detach from F-actin and it can be recruited to the synapse to induce functional changes.

CaMKII is a unique neuronal signaling protein that is composed of 12–14 subunits (for reviews [5,6]). In the brain, CaMKII predominantly consists of the α and β isoforms, which form heteromeric or homomeric complexes. CaMKIIα and β, like the two other isoforms, consist of four distinct domains: a catalytic domain containing the active site that is required for CaMKII kinase activity, a regulatory domain that comprises a self-inhibitory region and a binding site for the Ca^2+^/CaM complex, a variable domain and a hub or association domain necessary for assembly of the 12–14 subunits (Figure 1A). The kinase activity is regulated by the autoinhibitory regulatory segment, which blocks the substrate binding site in the absence of Ca^2+^. In response to an increase in intracellular Ca^2+^ concentration, Ca^2+^-bound calmodulin (Ca^2+^/CaM) competitively binds to the regulatory segment and it relieves inhibition by exposing the substrate-binding site (Figure 1B). This binding causes autophosphorylation at Thr286 (on CaMKIIα) or Thr287 (on CaMKIIβ) and it makes CaMKII activity Ca^2+^-independent [6].

Although the two main brain isoforms show similar domain structure and high sequence homology (89%–93% sequence homology in the catalytic and regulatory domains in rats) [7], they differ within the N-terminal part of the variable region, where CaMKIIβ, but not CaMKIIα, contains a filamentous actin (F-actin) binding domain (FABD) (Figure 1A). CaMKIIβ not only binds to actin [8], but it is also capable of bundling actin thanks to its actin-binding and association domains [9]. This bundling feature is achieved by the CaMKII oligomers binding to multiple actin filaments. It should be mentioned that the variable region, where CaMKIIα and β differ most, is subject to alternative splicing in all CaMKII isoforms. Regarding CaMKIIβ, four splicing variants, β, β’, βe, and β’e, were discovered in the brain, but only β and β’variants contain a FABD and are, therefore, able to bind to F-actin [10,11,12,13] (Figure 1A).

Besides actin, CaMKIIβ has been shown to specifically interact with some targets, but not CaMKIIα, such as Arc/Arg3.1 [14] or the centrosomal targeting protein PCM1 (pericentriolar material 1) [15]. In addition to these differences in susbstrate specificity, the two isoforms have different sensitivities to Ca^2+^ signals, since the binding affinity for calmodulin is higher for CaMKIIβ homomers than for CaMKIIα homomers [4]. Moreover, the rate of autophosphorylation is also more elevated for β than α [16]. 

### 1.2. CaMKIIβ Expression in the Nervous System 

Another important difference between the two major brain CaMKII isoforms is their temporal expression (Table 1). Indeed, CaMKIIβ is already expressed in the brain during embryonic life, starting around E12.5, whereas CaMKIIα starts to be expressed after birth and it becomes predominant in juvenile animals [3,17,18]. Regional differences also exist in the expression of the CaMKIIα and CaMKIIβ isoforms. For example, although they are both expressed in the forebrain and cerebellum, CaMKIIα is predominant in the adult hippocampus and neocortex [1,4,19], whereas CaMKIIβ is the dominant isoform in the cerebellum [1,3,19,20]. At the cellular level, CaMKIIα and CaMKIIβ are mainly expressed in excitatory pyramidal neurons in the cortex and hippocampus, but only CaMKIIβ is found in inhibitory interneurons in these regions [21,22,23]. In the cerebellum, CaMKIIα is only expressed in Purkinje cells, whereas CaMKIIβ is also present in granule cells [24]. At the neuron subcellular level, CaMKIIβ is localized in dendrites and particularly enriched in filopodia and mature spines [8]. In addition to neurons, CaMKIIβ is found in oligodendrocytes [25,26].

In the mature brain, the full-length variant CaMKIIβ predominate in most brain regions, except in the hypothalamus and brainstem where equal β and β’ are detected [11]. In the whole embryonic brain, βe predominate at E16 and E18 [11]. However, in the embryonic cerebral cortex, β and β’ proteins are dominant at E16.5 [18].

Although CaMKIIα and β have been simultaneously discovered at the beginning of the 80′s [27], CaMKIIβ has received far less attention. CaMKIIβ has often been relegated to a redundant role within heteromeric complexes or as a scaffold that is responsible for targeting CaMKII enzyme to F-actin [8]. Nevertheless, in the last years, several mutant constructs (Table 2) and mice (Table 3) have been developed and have allowed uncovering specific functions for CaMKIIβ in the brain. In the next two sections, we will review its roles in neuronal development and plasticity.

## 2. CaMKIIβ in Neuronal Development

Neuronal development is a sequential process from neuronal progenitor proliferation to synaptic integration. The failure of one of these developmental steps can heavily impact on subsequent brain formation and later function. Interestingly, gross histological examination of adult *Camk2b^−/−^* mice revealed no significant differences in overall brain structure [20,32,34], implying that CaMKIIβ might dispensable for normal brain development. However, further studies using overexpression or knockdown approaches in vitro or in vivo have suggested that CaMKIIβ might have multiple functions in the late steps of this process, in particular during neuronal migration, dendrite morphogenesis, and spine/synapse formation.

### 2.1. CaMKIIβ and Neuronal Migration

**Figure 2:**
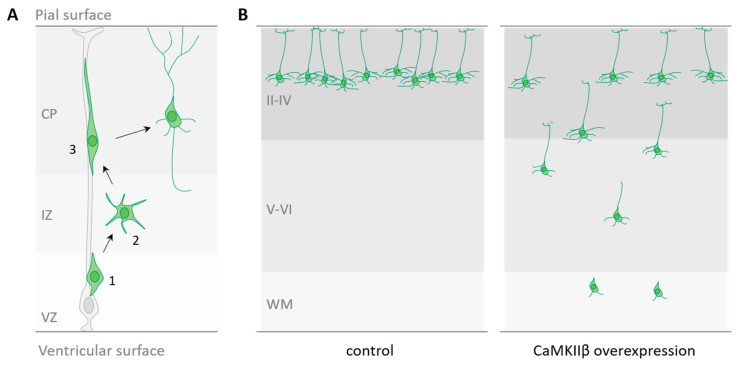
(**A**) Radial migration of projection neurons in the developing cerebral cortex. Step 1: newborn neurons detach from the ventricular surface. Step 2: nascent neurons migrate into the intermediate zone where they become transiently multipolar. Step 3: newborn neurons undergo a multipolar-bipolar transition and migrate along radial glia processes to finally detach and populate the cortical plate. VZ: ventricular zone; IZ: intermediate zone; CP: cortical plate. (**B**) Cortical neuron position in the early postnatal cortex in control condition of after CaMKIIβ overexpression. II-IV: layers II to IV; V-VI: layer V and VI; WM: white matter.

After their birth, neurons migrate to reach defined locations, where they integrate into functional circuits. Recently, we and others have shown that CaMKIIβ has a role during embryonic radial migration of cortical projections neurons [18,38]. Briefly, during the development of the cerebral cortex, projection neurons, born from progenitors in the germinal zone of the dorsal telencephalon, radially migrate following a route that is perpendicular to the ventricular surface before settling in their final laminar position [39]. Radial migration is a multi-step process that starts with the detachment of nascent neurons from the apical surface of the germinal ventricular zone (VZ) (Figure 2A step 1). Newly generated neurons then move to the intermediate zone (IZ), where they acquire a multipolar shape (Figure 2A step 2). Thereafter, neurons become bipolar, extending a leading process towards the pial surface and a trailing process in the opposite direction. Upon multi to bipolar transition, neurons establish dynamic contacts with radial glia fibers and subsequently use them as a scaffold for migrating to the upper part of the cortical plate (CP) using a mode of migration called locomotion (Figure 2A step 3). 

Using knockdown or overexpression approaches via in utero electroporation, correct levels of CaMKIIβ have been shown to be required for proper radial migration of projection neurons in two independent studies [18,38]. While CaMKIIβ overexpression impairs this process in both studies, opposite results have been described after electroporation of the mouse cerebral cortex with CaMKIIβ shRNAs. Indeed, after electroporation at E14.5 of a pool of five different CaMKIIβ shRNAs (targeting CaMKIIβ, but not α), Kury at al. observed migration defects at P0. While control electroporated neurons are located in the upper part of the CP at this stage, CaMKIIβ-silenced neurons are scattered throughout the entire cortical wall in this study [38]. Inversely, Nicole et al. found that the knockdown of CaMKIIβ at E14.5, with one specific shRNA (targeting only CaMKIIβ, but not α, γ, and δ, and targeting the four splice variants of CaMKIIβ), promotes the radial migration of cortical neurons. When CaMKIIβ is silenced, neurons quickly leave the IZ after the multipolar–bipolar transition to reach the CP and then CaMKIIβ-deficient cells move faster in the CP. Consequently, at E17.5, more cells reach the upper part of the CP compared to control neurons [18]. This discrepancy between the results might be due to off-target effects, a lack of specificity of the shRNAs towards the CaMKIIβ isoform, and/or to a variable action of these shRNA on the different splicing variants.

In the study of Nicole and colleagues, CaMKIIβ overexpression at E14.5 significantly decreases the proportion of cells reaching the CP three days later, whereas the fraction in the IZ is concomitantly increased. At P14, a significant fraction of CaMKIIβ overexpressing cells is still trapped in deep layers, indicating that this manipulation during embryogenesis has a long-term impact on neuronal positioning [18] (Figure 2B). In particular, CaMKIIβ has been found to be essential for the multipolar-bipolar transition and for locomotion in the CP, and these actions are primarily dependent on its actin-binding and oligomerization domains [18]. Indeed, the overexpression of CaMKIIβ-ΔFABD or CaMKIIβ-Δasso abolishes the capacity of CaMKIIβ to impair the multipolar–bipolar conversion and to reduce the migration speed in the CP [18]. Moreover, electroporation of the phosphomimetic All D mutant (which is unable to bind to F-actin, see Table 1) also does not induce any migration defect, indicating that autophosphorylation sites within the FABD control CaMKIIβ action in migrating neurons [18]. This action is also tightly linked to cofilin activity since F-actin–CaMKIIβ interaction limits access of actin regulating proteins, like the actin-depolymerizing cofilin [18,30]. Interestingly, Ca^2+^ fluctuations have been described in migrating neurons [40,41] and it has been proposed that CaMKIIβ might constitute a link between Ca^2+^ signaling and the actin cytoskeleton in cortical migrating neurons. Thus Ca^2+^ increase in locomoting neurons might dissociate CaMKIIβ from F-actin allowing for actin remodeling by actin-modifying proteins, such as cofilin, and thus the forward movement [18]. Finally, while the actin-binding and -bundling activities of CaMKIIβ seem to be primarily involved in the regulation of cortical neuron migration, its kinase activity might also be implicated, since mutations that decrease or increase CaMKIIβ auto-phosphorylation at Thr287 also affect migration in the developing cortex [38].

In contrast to the β isoform, CaMKIIα is not expressed in the embryonic cerebral cortex [3,17,18], and reducing or increasing levels of wild-type CaMKIIα does not affect cortical neuron migration [38]. Accordingly, CaMKIIβ has a role in the radial migration of cortical projection neurons, but not CaMKIIα.

### 2.2. CaMKIIβ and Dendrite Formation/Pruning

Once newborn neurons reach their final destination, they undergo dendrite morphogenesis with a first step of dendrite growth and arborization followed by a second step of dendrite retraction and pruning [42]. These steps are crucial for establishing accurate dendrite morphologies and, consequently, proper neural circuits in the brain.

The role of CaMKIIβ in this process has been well described in granule neurons of the developing cerebellar cortex [15]. In vitro, the knockdown of CaMKIIβ in these neurons induces more and longer dendrites, but has no effect on axon length, demonstrating that CaMKIIβ specifically inhibits dendrite growth and arborization [15]. Similarly, in vivo, CaMKIIβ silenced granule neurons exhibit longer dendrites with greater secondary and tertiary dendrite branching as compared to control neurons at P8, five days after the electroporation. At P12, while control neurons have a few short dendrites with simplified arbors compared to P8, CaMKIIβ-knockdown neurons show longer, more branched dendrite arbors with greater total dendrite length and dendrite number when compared to controls indicating that CaMKIIβ silencing also impairs dendrite pruning [15]. Interestingly, CaMKIIβ drives dendrite retraction and pruning from the centrosome. Puram et al. identified a unique centrosomal targeting sequence (CTS) within the variable region of CaMKIIβ (but not CaMKIIα). This CTS mediates the specific interaction of CaMKIIβ with the centrosomal targeting protein PCM1, which then induces the localization of CaMKIIβ to the centrosome. There, CaMKIIβ phosphorylates the E3 ubiquitin ligase Cdc20-APC (cell division cycle 20–anaphase promoting complex) at Ser51, which induces Cdc20 dispersion from the centrosome, thereby inhibiting centrosomal Cdc20-APC activity and triggering a switch from growth to retraction of dendrites [15]. In this study, the authors also demonstrate that CaMKIIβ operates at the centrosome in a CaMKIIα-independent manner, thus unrevealing another isoform specific-function for CaMKIIβ. This function of CaMKIIβ in the regulation of dendrite retraction has been also described in cultured hippocampal and cortical neurons [15] as well as, in vivo, in pyramidal cortical neurons and a similar mechanism seems to be involved in these cells [18]. Thus, CaMKIIβ restricts the growth and arborization of dendrites in diverse populations of mammalian brain neurons. However, it should be noted that this function might be cell specific, since the complexity of dendritic branching is not significantly changed in Purkinje cells of *Camk2b^−/−^* mutants as compared to the control mice [20].

### 2.3. CaMKIIβ and Spine/Synapse Formation

While dendrites develop, they are progressively covered by small protrusions, called dendritic spines. These spines serve as the main sites of excitatory synapses in the brain. During the early stages of synaptogenesis, immature dendritic protrusions, which are classified as filopodia, rapidly protrude and retract from dendrites, allowing for neurons to find contact sites, which can then evolve into synapses. With development, filopodia are gradually replaced by mature mushroom-shaped spines [43]. Importantly, the actin filaments are a major structural element of the regulation of dendritic spine formation and morphology [44].

In young hippocampal cultured neurons, Fink et al. showed that CaMKIIβ overexpression increases the fine architecture of the dendritic arbor in particular filopodia, whereas its knockdown has an opposite effect [28]. However, this ability of CaMKIIβ to promote dendritic arborization decreases with age and its action becomes even opposite when neurons mature [28]. As neurons mature, CaMKIIβ controls the motility of filopodia and synapse formation rather positively [28]. Again, only CaMKIIβ has this activity, not CaMKIIα, most probably because it is dependent on CaMKIIβ ability to interact with F-actin [28]. Besides its role during spinogenesis, CaMKIIβ is also crucial for spine maintenance [9]. Indeed, CaMKIIβ maintains mature spine structure through its F-actin binding and bundling activity, but not its kinase activity [9].

Similarly, in Purkinje cells, CaMKIIβ promotes spine formation and elongation via its F-actin bundling activity [45]. In addition, protein kinase C (PKC)-mediated phosphorylation of CaMKIIβ is responsible for the maintenance of the appropriate spine density and morphology in these cells. More precisely, PKC phosphorylates CaMKIIβ at S315 under the control of group I metabotropic glutamate receptor (mGluR1) signaling, and this event results in dissociation of the CaMKIIβ/F-actin complex, which then represses excessive spine formation and elongation in mature Purkinje cells [45].

While these data suggest that CaMKIIβ is important for the control of spine density and morphology, other studies give somehow different conclusions. For example, Okamoto et al. observed that, after CaMKIIβ knockdown in hippocampal organotypic slices, mature spines are converted to filopodia-like spines, but spine density is not modified [9]. Furthermore, in *Camk2b^−/−^* mice, Purkinje cells and hippocampal pyramidal cells from CA1 do not show any differences in spine density and/or morphology [20,34].

Although the initial observations in *Camk2b^−/−^* mutants suggested that CaMKIIβ is not required for normal neuronal development, the aforementioned studies inversely indicate that CaMKIIβ seems to have several functions during this process. The strategy used to perturb CaMKIIβ expression (knockdown, knockout, surexpression, in vitro/in vivo...) might explain the distinct conclusions. Indeed, germline mutations in *Camk2b* might result in homeostatic compensatory mechanisms that would prevent to see the changes that were observed in neurons shortly after an acute depletion in a limited population. 

## 3. CaMKIIβ in Neuronal Plasticity

One fundamental attribute of the brain is the plasticity of its synapses, namely a positive or negative change in efficacy of connections between neurons in response to neuronal activity. Depending on the specific pattern of stimulation and localization of neuron assemblies, individual synapses can increase or decrease the strength of their transmission, two processes called, respectively, long term potentiation (LTP) and long term depression (LTD). These changes in synapse functioning have been considered as a cellular model for the process of learning and memory (for review [46]). It is now well established that LTP induction results in Ca^2+^ entry, which activates CaMKIIα localized close to the activated synapse. Subsequently, CaMKIIα translocates to the synapse, where it binds to N-Methyl-D-aspartate (NMDA) receptors and produces the potentiation of the synaptic response by phosphorylating principal and auxiliary subunits of α-amino-3-hydroxy-5-methyl-4-isoxazolepropionic acid (AMPA) receptors (for review [47]). This sequestration of CaMKIIα to dendritic spines and the postsynaptic density (PSD) within a few seconds of stimulation is coupled to actin polymerization and the expansion of the stimulated spines [48], also called structural plasticity [49]. Blocking structural spine enlargement interferes with functional plasticity induction [30], which suggests that the functional and structural plasticity are tightly and mutually regulated. These structural and functional changes are short-lasting (1–4 h), unless stabilizing plasticity-related proteins (PRPs) are recruited. This recruitment depends on new protein synthesis and on a process of “synaptic tagging and capture”, which explain how the newly synthesized proteins in the soma can selectively find the potentiated synapses (for review [50]). The postsynaptic capture of PRPs allows for the subsequent stabilization of spine structure, which enables maintenance of the functional change. This synaptic tagging process at the dendritic spine requires CaMKII activity and the newly formed F-actin complex that is both permissive and necessary for the remodeling of the PSD. Among the PRPs, Arc is rapidly upregulated by strong synaptic activity and it critically contributes to weakening adjacent synapses by promoting AMPA receptor endocytosis to prevent undesired enhanced activity in the vicinity of activated synapses. This process that is necessary for sustainably changing synaptic efficiency only on potentiated synapses is called “inverse synaptic tagging” [14].

In this whole complex cascade, CaMKIIβ acts in concert with CaMKIIα, but with distinct functions to regulate both functional and structural plasticity and the synaptic tagging process, necessary for learning and memory.

### 3.1. Molecular Mechanisms of CaMKIIβ in Synaptic Plasticity

The C-terminal association domain of CaMKII isoforms can form homo- or heteromeric assemblies of 12 to 14 mers, in which subunit composition seems to be dependent on isoform expressions (with unknown isoform preference). In the forebrain, the β subunit of CaMKII constitutes approximately 30% of the total amount of CaMKII [19] and 80% in the cerebellum. As a consequence, the ratios of α and β subunits are about 3:1 and 1:4 in adult forebrain and cerebellum, respectively [19]. In the holoenzyme, the presence of CaMKIIβ plays a critical role in the subcellular localization and subsequent postsynaptic translocation of the entire CaMKII holozyme [8,29]. Indeed, in the basal condition, the CaMKII holoenzymes, via CaMKIIβ, are able to bind actin filaments, particularly in dendritic spines [8,9,28,29,48]. When CaMKII holoenzyme is activated by neuronal activity and the resultant Ca^2+^ influx, it detaches from F-actin and CaMKII holoenzymes can be recruited to the synapse to induce functional changes (Figure 1B). Thus, a small fraction of CaMKIIβ is sufficient for docking the predominant CaMKIIα to the actin cytoskeleton. In this configuration, CaMKIIβ functions as a “targeting module” that localizes a higher number of CaMKIIα to synaptic sites of action in order to facilitate functional plasticity [8].

In addition, the interaction of CaMKIIβ with F-actin in basal state [9] limits the binding of other actin-binding molecules [30]. During neuronal plasticity, the CaMKIIβ/actin interaction is abolished by Ca^2+^/CaM binding [8], which results in the detachment and release of unbundled F-actin. This opens a brief time window during which other actin binding partners have access to F-actin and can profoundly remodel the filaments [51,52]. The subsequent inactivation of CaMKIIβ results in the re-bundling of the polymerized F-actin and re-stabilization of the new dendritic spine structure. Blocking the detachment of CaMKIIβ from F-actin without affecting the kinase activity results in a deficit of both structural and functional plasticity [30], highlighting the crucial dual function of CaMKIIβ as a negative regulator of actin remodeling in spines and as a molecular-temporal gate of synaptic plasticity. 

More recently, a potential role of a dynamic interaction between inactive CaMKIIβ and Arc has been shown. This interaction would allow Arc to be preferentially maintained at inactive synapses rather than active synapses. CaMKII-stabilized Arc may efficiently contribute to promoting AMPA receptor clearance from the inactive synapses. In this “inverse synaptic tagging process”, CaMKIIβ acts as a scaffold for Arc in dendritic spines [14]. In addition, it has been recently suggested that local CaMKIIα translation at activated synapses [53,54] and subunit exchange inside the CaMKII holoenzyme that is triggered by CaMKII activation [55,56] would aid local replacement of the β isoform in CaMKII holoenzyme, releasing CaMKIIβ to the neighboring non-potentiated synapses [5].

### 3.2. Role of CaMKIIβ in LTP (Hippocampal and Cerebellar)

In contrast to numerous studies on CaMKIIα, the analysis of the role that is played by CaMKIIβ in LTP has been carried on in limited experiments. By selectively increasing CaMKIIβ expression in the dentate gyrus (DG), the group of J. Tsien has demonstrated that elevated CaMKIIβ activity leads to reduced LTP in the perforant path of the DG [57]. However, the inducible chemical genetic method used also increases the β/α ratio and changes the composition of the CaMKII holoenzyme. Because CaMKIIβ has an F-actin binding module, which confers the anchoring of the CaMKII complex at the base of the synaptic spine, the numerical increase of CaMKIIβ in CaMKII holoenzyme might alter the biophysical properties of the holoenzyme (i.e., the CaMKII holoenzyme might be slower to translocate from the base of dendritic spines to PSD or to fall off faster from PSD). In contrast, by using the *Camk2b^−/−^* mutant, Borgesius et al. (2011) clearly established that Schaffer collateral-CA1 LTP is highly dependent upon the presence of CaMKIIβ. But interestingly LTP was unaffected in *Camk2b^A303R^* mutant, in which the Ca2+/CaM-dependent activation of CaMKIIβ is prevented, while the F-actin binding and bundling property are preserved. This study pinpoints that CaMKIIβ can modulate LTP via a non-enzymatic role, as a targeting module of CaMKIIα. More recently, the relative importance of CaMKIIα and CaMKIIβ in LTP has been analyzed while using a CRISPR-based system to delete both CaMKII α and β (DKO) and using rescue experiments to restore the defects that are caused by the DKO by expressing back CaMKII subunits [58]. First, these authors confirmed that the deletion of CaMKIIβ blocks LTP, similar to the deletion of CaMKIIα. However, interestingly, while CaMKIIα fully rescued LTP in the DKO, CaMKIIβ was unable to rescue LTP, either in the DKO or after CaMKIIα deletion alone, indicating that CaMKIIβ is not required for the full expression of LTP.

Since CaMKIIβ is highly expressed in the cerebellum, its role has also been studied in cerebellar synaptic plasticity. The plasticity rules are different in the cerebellum when compared to the hippocampus. The stimulation of parallel fibers in combination with climbing fibers results in a high influx of Ca^2+^ and the recruitment of CaMKIIα, resulting in LTD at the parallel fiber-Purkinje cell synapses [59]. Interestingly, in *Camk2b^−/^* mutant mice^−^, the loss of CaMKIIβ results in a complete reversal of the plasticity rules [20]. A protocol that induced synaptic depression in wild type mice resulted in synaptic potentiation in *Camk2b* knock-out-mice and vice-versa. Although the precise mechanism is unclear, a mathematical model that was recently developed suggests that the balance of CaMKII-mediated phosphorylation and protein phosphatase 2B (PP2B)-mediated dephosphorylation of AMPA receptors can determine whether LTD or LTP occurs in cerebellar purkinje cells. This computational model replicates observations that CaMKIIβ controls the direction of plasticity. It also demonstrates that the binding of F-actin to CaMKII can enable the β isoform of the kinase to regulate bidirectional plasticity at these synapses [60].

### 3.3. CaMKIIβ and Memory

Based on the role of CaMKIIβ in LTP, studies have been also conducted in order to understand the specific role of CaMKIIβ in learning and memory processes. Increased activity of CaMKIIβ selectively in the DG, using the inducible chemical genetic model, induces a reversal learning deficits in the radial arm maze task and the water cross maze [61], but a normal memory formation, one day after training in novel object recognition, and contextual fear memory. However, their contextual fear memory is severely impaired at longer retention delays (10 days) [57], which suggests that the β isoform could have an effect in long term memory consolidation. On the other hand, *Camk2b^−/−^* mutant mice show impairment in novel object recognition when tested 4 h after training [32] and in fear conditioning memory when tested 24 h after training [34]. Interestingly, in this last work, the authors found that CaMKIIα expression levels are not altered in the *Camk2b^−/−^* mice but its location to synapses is decreased by almost 40%. This prompted the authors to study whether the observed memory deficits were a side-effect of decreased CaMKIIα localization in PSD. To test this, they used the *Camk2b^A303R^* mouse model and showed that fear conditioning performance was normal, in contrast to what they observed in *Camk2b^−/−^* mice. CaMKIIα abnormal distribution observed in *Camk2b^−/−^* was not seen in *Camk2b^A303R^* mice. This last result led to the important conclusion that CaMKIIβ binding to F-actin is necessary for CaMKIIα translocation to spines, but not its catalytic activity, and consequently for memory processes. The discrepancy between observations that are based on knock-in mouse lines versus overexpression of mutant subunits might be also due to the relative stoichiometry of CaMKIIβ and CaMKIIα, which might influence CaMKIIα interactions in the PSD. Indeed, Silva et colleagues have reported that, whereas the heterozygous *Camk2a^−^/^+^* (with a probably higher β/α ratio) exhibit normal learning and memory after 1–3 days of retention, these mice show a severe impairment of retention of long term memory at 10–50 days [62], as observed with the chemical genetic method that increase CaMKIIβ (with a probably higher β/α ratio) [57] Thus, a higher content of CaMKIIβ within the holoenzymes might cause the CaMKII complex to be slower to translocate to the PSD. At the opposite, the lack of CaMKIIβ could reduce the synaptic location of CaMKIIα and also alter the bioavailability closed to the PSD. Altogether, these results strongly suggest an essential, but non-enzymatic role for CaMKIIβ in learning and memory, most probably by properly targeting the CAMKII complex.

## 4. CaMKIIβ and Brain Disorders

As a consequence of its important physiological roles in neuronal development and plasticity, alterations in CaMKIIβ expression/function could contribute to the pathogenesis of many brain disorders. In this vein, a series of recent studies suggest that CaMKIIβ dysfunction in the brain may underlie multiple neuropsychiatric and neurodevelopmental disorders. 

In 2017, Kury et al. identified, via a whole-exome sequencing approach, seven rare *de novo CAMK2B* variants in 10 unrelated individuals with mild to severe intellectual disability [38]. These individuals also show language impairment and behavioral anomalies, such as abnormal emotion/affect behavior or seizures for some of them. Interestingly, most of the described mutations decrease or increase CaMKIIβ auto-phosphorylation at Thr287 and the expression of the corresponding mutant forms of CaMKIIβ in the mouse developing cerebral cortex affect neuronal migration [38]. Few months later, another collaborative study published two other *de novo* variants in *CAMK2B* that impair the autoinhibition of CaMKII. These mutations were identified in two individuals amongst a population of 976 individuals with intellectual disability, developmental delay, and epilepsy [63]. 

Several converging evidence also support a potential role of CaMKIIβ in the pathophysiology of schizophrenia. Indeed, the prefrontal cortex (a key brain region that is involved in the cognitive symptoms of the disease) of patients who had schizophrenia shows elevated levels of CaMKIIβ transcripts [64,65]. Moreover, an increase of CaMKIIβ mRNA was found in several animal models of schizophrenia, such as postnatal maternal deprivation and pubertal stress [66], as well as amphetamine sensitization [67]. 

In addition to neurodevelopmental disorders, CaMKIIβ might be a molecular determinant of depression. Indeed, its expression is increased at the mRNA level in the human frontal cortex of depression tissues [65] and, at the protein level, CaMKIIβ is significantly upregulated in the lateral habenula (nucleus that has emerged as a key brain region in aversive behaviors and the pathophysiology of depression) of animal models of depression and down-regulated by antidepressants [68]. Similarly, in the hippocampal CA1, CaMKIIβ is significantly upregulated in depressed rats, while antidepressant treatment downregulated this protein [69]. Furthermore, increasing CaMKIIβ in the lateral habenula through a viral approach is sufficient for producing profound depressive symptoms, including anhedonia and behavioral despair, in both rats and mice [68]. To note, the overexpression of CaMKIIα at a similar infection rate does not cause similar depressive-like effects. Conversely, down-regulation of CaMKIIβ levels in this structure, blocking its activity or its target molecule the glutamate receptor GluR1 reverse the depressive symptoms [68]. A similar manipulation of CaMKIIβ (overexpression and downregulation) in the CA1 gives similar results on depressive-like behavior [69]. CaMKIIβ seems to act upstream of the cyclo-oxygenase (COX)-2/prostaglandin E2 (PGE2) neuroinflammatory signaling pathway in this hippocampal region [69]. 

## 5. Conclusions

The recent findings on CaMKIIβ has demonstrated that this isoform has major biological functions in the brain, and it might be a potential target for therapeutic interventions in diverse brain disorders. However, further studies are required to better define the spatiotemporal and subcellular functions of CaMKIIβ, but also to provide mechanistic insights into CaMKIIβ action. A better understanding of the role(s) played by the individual CaMKIIβ splice variants represents another important challenge. Finally, it would now be interesting to consider CaMKIIγ and δ, which are able to bind to F-actin [70], but have been also understated.

## Figures and Tables

**Table 1:** Schematic representation of temporal and regional expression of CaMKIIα and CaMKIIβ.

		CaMKIIα	CaMKIIβ
Temporal expression (total brain)	Embryonic life	− ^2^	+ ^3^
	Post-natal	+	++
	Adult	+++	++
Regional expression (adult brain)	Hippocampus	+++	+
	Cerebral cortex	+++	+
	Cerebellum	+	++++
Cellular expression	Excitatory pyramidal neurons ^1^	+	+
	Inhibitory interneurons ^1^	−	+
	Purkinje cells	+	+
	Cerebellar granule neurons	−	+

^1^ In cerebral cortex and hippocampus; ^2^ − indicates not expressed; ^3^ + indicates expressed.

**Table 2:** CaMKIIβ mutants.

Name	Description	References
CaMKIIβ-T287D	Constitutively active mutant	[12,17,28,29]
CaMKIIβ-A303R	Ca2^+^/CaM binding-deficient mutant	[12,17,28,29]
CaMKIIβ-K43R	Impaired for ATP binding, kinase inactive mutant	[9,12,28,29]
CaMKIIβ285-542 CaMKIIβ344-542	Mutants lacking kinase domain but containing F-actin binding and association domains	[9]
CaMKIIβ-ΔFABD	Mutant without F-actin binding domain	[15,18]
CaMKIIβ1-401	Mutant without association domain	[9]
CaMKIIβ-Δasso	Mutant without association domain	[15,18]
CaMKIIβ285-401	Mutant without kinase and association domains	[9]
CaMKIIβ-ΔCTS	Mutant without centrosomal targeting sequence	[15,18]
CaMKIIβ-All A	Phosphoblock All A mutant (all S and T residues within the FABD are changed to alanine)	[18,30]
CaMKIIβ-All D	Phosphomimetic All D mutant (all S and T residues within the FABD are changed to aspartic acid), loss of F-actin-binding activity	[18,30]

**Table 3:** *Camk2b* mutant mice.

Name	Description	References
*Camk2b^−/−^*	Deletion exon 11	[20]
*Camk2b^−/−^*	Deletion exon 2	[31]
*Camk2b^−/−^*	*Camk2b^f^/^f^* (loxP sites flanking exons 7–8) crossed with a CMV-Cre mouse	[32]
*Camk2b^f^/^f^*	LoxP sites flanking exon 2	[33]
*Camk2b^A303R^/^A303R^*	Mutant which cannot bind Ca^2+^/CaM (mutation prevents CaMKIIβ enzymatic activation, while preserving its ability to bind to actin)	[34]
*Camk2b^T287A^/^T287A^*	Autophosphorylation-deficient mutant (mutation blocks CaMKIIβ autonomous activity)	[33]
*Camk2b exon 13:TS/A knock-in mouse*	Mouse carrying phosphoblock mutations in the actin binding domain (phosphorylation sites of this region are critical for CaMKII detachment from F-actin)	[35]
*Camk2a^−/−^;Camk2b* ^−/−^	*Camk2a^−/−^* [36] and *Camk2b^−/−^* [20] were used to generate double mutants	[37]

## Abbreviations

AMPA	α-amino-3-hydroxy-5-methyl-4-isoxazolepropionic acid
Ca^2+^/CaM	Ca^2+^-bound calmodulin
CaMKII	calcium/calmodulin-dependent protein kinase II
Cdc20-APC	cell division cycle 20–anaphase promoting complex
COX-2	cyclo-oxygenase-2
CP	cortical plate
CTS	centrosomal targeting sequence
DG	dentate gyrus
DKO	deletion of both CaMKIIα and CaMKIIβ
FABD	F-actin binding domain
F-actin	filamentous-actin
IZ	intermediate zone
LTD	long-term depression
LTP	long-term potentiation
mGluR1	group I metabotropic glutamate receptor
NMDA	N-Methyl-D-aspartate
PCM1	pericentriolar material 1
PGE2	prostaglandin E2
PKC	protein kinase C
PP2B	protein phosphatase 2B
PRP	plasticity-related proteins
PSD	post-synaptic density
VZ	ventricular zone
WM	white matter

## References

[1] Erondu N.E., Kennedy M.B. (1985). Regional distribution of type II Ca2+/calmodulin-dependent protein kinase in rat brain. J. Neurosci. Off. J. Soc. Neurosci..

[2] Tombes R.M., Faison M.O., Turbeville J.M. (2003). Organization and evolution of multifunctional Ca(2+)/CaM-dependent protein kinase genes. Gene.

[3] Bayer K.U., Lohler J., Schulman H., Harbers K. (1999). Developmental expression of the CaM kinase II isoforms: Ubiquitous gamma- and delta-CaM kinase II are the early isoforms and most abundant in the developing nervous system. Brain Res. Mol. Brain Res..

[4] Brocke L., Chiang L.W., Wagner P.D., Schulman H. (1999). Functional implications of the subunit composition of neuronal CaM kinase II. J. Biol. Chem..

[5] Bayer K.U., Schulman H. (2019). CaM Kinase: Still Inspiring at 40. Neuron.

[6] Hell J.W. (2014). CaMKII: Claiming center stage in postsynaptic function and organization. Neuron.

[7] Tobimatsu T., Fujisawa H. (1989). Tissue-specific expression of four types of rat calmodulin-dependent protein kinase II mRNAs. J. Biol. Chem..

[8] Shen K., Teruel M.N., Subramanian K., Meyer T. (1998). CaMKIIbeta functions as an F-actin targeting module that localizes CaMKIIalpha/beta heterooligomers to dendritic spines. Neuron.

[9] Okamoto K., Narayanan R., Lee S.H., Murata K., Hayashi Y. (2007). The role of CaMKII as an F-actin-bundling protein crucial for maintenance of dendritic spine structure. Proc. Natl. Acad. Sci. USA.

[10] Brocke L., Srinivasan M., Schulman H. (1995). Developmental and regional expression of multifunctional Ca2+/calmodulin-dependent protein kinase isoforms in rat brain. J. Neurosci. Off. J. Soc. Neurosci..

[11] Cook S.G., Bourke A.M., O’Leary H., Zaegel V., Lasda E., Mize-Berge J., Quillinan N., Tucker C.L., Coultrap S.J., Herson P.S. (2018). Analysis of the CaMKIIalpha and beta splice-variant distribution among brain regions reveals isoform-specific differences in holoenzyme formation. Sci. Rep..

[12] O’Leary H., Lasda E., Bayer K.U. (2006). CaMKIIbeta association with the actin cytoskeleton is regulated by alternative splicing. Mol. Biol. Cell.

[13] Zheng J., Redmond L., Xu C., Kuang J., Liao W. (2014). Alternative splicing in the variable domain of CaMKIIbeta affects the level of F-actin association in developing neurons. Int. J. Clin. Exp. Pathol..

[14] Okuno H., Akashi K., Ishii Y., Yagishita-Kyo N., Suzuki K., Nonaka M., Kawashima T., Fujii H., Takemoto-Kimura S., Abe M. (2012). Inverse synaptic tagging of inactive synapses via dynamic interaction of Arc/Arg3.1 with CaMKIIbeta. Cell.

[15] Puram S.V., Kim A.H., Ikeuchi Y., Wilson-Grady J.T., Merdes A., Gygi S.P., Bonni A. (2011). A CaMKIIbeta signaling pathway at the centrosome regulates dendrite patterning in the brain. Nat. Neurosci..

[16] Gaertner T.R., Kolodziej S.J., Wang D., Kobayashi R., Koomen J.M., Stoops J.K., Waxham M.N. (2004). Comparative analyses of the three-dimensional structures and enzymatic properties of alpha, beta, gamma and delta isoforms of Ca2+-calmodulin-dependent protein kinase II. J. Biol. Chem..

[17] Lin Y.C., Redmond L. (2008). CaMKIIbeta binding to stable F-actin in vivo regulates F-actin filament stability. Proc. Natl. Acad. Sci. USA.

[18] Nicole O., Bell D.M., Leste-Lasserre T., Doat H., Guillemot F., Pacary E. (2018). A novel role for CAMKIIbeta in the regulation of cortical neuron migration: Implications for neurodevelopmental disorders. Mol. Psychiatry.

[19] Miller S.G., Kennedy M.B. (1985). Distinct forebrain and cerebellar isozymes of type II Ca2+/calmodulin-dependent protein kinase associate differently with the postsynaptic density fraction. J. Biol. Chem..

[20] van Woerden G.M., Hoebeek F.E., Gao Z., Nagaraja R.Y., Hoogenraad C.C., Kushner S.A., Hansel C., De Zeeuw C.I., Elgersma Y. (2009). betaCaMKII controls the direction of plasticity at parallel fiber-Purkinje cell synapses. Nat. Neurosci..

[21] Liu X.B., Jones E.G. (1996). Localization of alpha type II calcium calmodulin-dependent protein kinase at glutamatergic but not gamma-aminobutyric acid (GABAergic) synapses in thalamus and cerebral cortex. Proc. Natl. Acad. Sci. USA.

[22] Sik A., Hajos N., Gulacsi A., Mody I., Freund T.F. (1998). The absence of a major Ca2+ signaling pathway in GABAergic neurons of the hippocampus. Proc. Natl. Acad. Sci. USA.

[23] Thiagarajan T.C., Piedras-Renteria E.S., Tsien R.W. (2002). alpha- and betaCaMKII. Inverse regulation by neuronal activity and opposing effects on synaptic strength. Neuron.

[24] Conlee J.W., Shapiro S.M., Churn S.B. (2000). Expression of the alpha and beta subunits of Ca2+/calmodulin kinase II in the cerebellum of jaundiced Gunn rats during development: A quantitative light microscopic analysis. Acta Neuropathol..

[25] Cahoy J.D., Emery B., Kaushal A., Foo L.C., Zamanian J.L., Christopherson K.S., Xing Y., Lubischer J.L., Krieg P.A., Krupenko S.A. (2008). A transcriptome database for astrocytes, neurons, and oligodendrocytes: A new resource for understanding brain development and function. J. Neurosci. Off. J. Soc. Neurosci..

[26] Waggener C.T., Dupree J.L., Elgersma Y., Fuss B. (2013). CaMKIIbeta regulates oligodendrocyte maturation and CNS myelination. J. Neurosci. Off. J. Soc. Neurosci..

[27] Bennett M.K., Erondu N.E., Kennedy M.B. (1983). Purification and characterization of a calmodulin-dependent protein kinase that is highly concentrated in brain. J. Biol. Chem..

[28] Fink C.C., Bayer K.U., Myers J.W., Ferrell J.E., Schulman H., Meyer T. (2003). Selective regulation of neurite extension and synapse formation by the beta but not the alpha isoform of CaMKII. Neuron.

[29] Shen K., Meyer T. (1999). Dynamic control of CaMKII translocation and localization in hippocampal neurons by NMDA receptor stimulation. Science.

[30] Kim K., Lakhanpal G., Lu H.E., Khan M., Suzuki A., Hayashi M.K., Narayanan R., Luyben T.T., Matsuda T., Nagai T. (2015). A Temporary Gating of Actin Remodeling during Synaptic Plasticity Consists of the Interplay between the Kinase and Structural Functions of CaMKII. Neuron.

[31] Gao Z., van Woerden G.M., Elgersma Y., De Zeeuw C.I., Hoebeek F.E. (2014). Distinct roles of alpha- and betaCaMKII in controlling long-term potentiation of GABAA-receptor mediated transmission in murine Purkinje cells. Front. Cell. Neurosci..

[32] Bachstetter A.D., Webster S.J., Tu T., Goulding D.S., Haiech J., Watterson D.M., Van Eldik L.J. (2014). Generation and behavior characterization of CaMKIIbeta knockout mice. PLoS ONE.

[33] Kool M.J., van de Bree J.E., Bodde H.E., Elgersma Y., van Woerden G.M. (2016). The molecular, temporal and region-specific requirements of the beta isoform of Calcium/Calmodulin-dependent protein kinase type 2 (CAMK2B) in mouse locomotion. Sci. Rep..

[34] Borgesius N.Z., van Woerden G.M., Buitendijk G.H., Keijzer N., Jaarsma D., Hoogenraad C.C., Elgersma Y. (2011). betaCaMKII plays a nonenzymatic role in hippocampal synaptic plasticity and learning by targeting alphaCaMKII to synapses. J. Neurosci. Off. J. Soc. Neurosci..

[35] Kim K., Suzuki A., Kojima H., Kawamura M., Miya K., Abe M., Yamada I., Furuse T., Wakana S., Sakimura K. (2019). Autophosphorylation of F-actin binding domain of CaMKIIbeta is required for fear learning. Neurobiol. Learn. Mem..

[36] Elgersma Y., Fedorov N.B., Ikonen S., Choi E.S., Elgersma M., Carvalho O.M., Giese K.P., Silva A.J. (2002). Inhibitory autophosphorylation of CaMKII controls PSD association, plasticity, and learning. Neuron.

[37] Kool M.J., Proietti Onori M., Borgesius N.Z., van de Bree J.E., Elgersma-Hooisma M., Nio E., Bezstarosti K., Buitendijk G.H.S., Aghadavoud Jolfaei M., Demmers J.A.A. (2019). CAMK2-Dependent Signaling in Neurons Is Essential for Survival. J. Neurosci. Off. J. Soc. Neurosci..

[38] Kury S., van Woerden G.M., Besnard T., Proietti Onori M., Latypova X., Towne M.C., Cho M.T., Prescott T.E., Ploeg M.A., Sanders S. (2017). De Novo Mutations in Protein Kinase Genes CAMK2A and CAMK2B Cause Intellectual Disability. Am. J. Hum. Genet..

[39] Azzarelli R., Guillemot F., Pacary E. (2015). Function and regulation of Rnd proteins in cortical projection neuron migration. Front. Neurosci..

[40] Komuro H., Rakic P. (1996). Intracellular Ca2+ fluctuations modulate the rate of neuronal migration. Neuron.

[41] Rash B.G., Ackman J.B., Rakic P. (2016). Bidirectional radial Ca(2+) activity regulates neurogenesis and migration during early cortical column formation. Sci. Adv..

[42] Puram S.V., Bonni A. (2013). Cell-intrinsic drivers of dendrite morphogenesis. Development.

[43] Hering H., Sheng M. (2001). Dendritic spines: Structure, dynamics and regulation. Nat. Rev. Neurosci..

[44] Hlushchenko I., Koskinen M., Hotulainen P. (2016). Dendritic spine actin dynamics in neuronal maturation and synaptic plasticity. Cytoskeleton.

[45] Sugawara T., Hisatsune C., Miyamoto H., Ogawa N., Mikoshiba K. (2017). Regulation of spinogenesis in mature Purkinje cells via mGluR/PKC-mediated phosphorylation of CaMKIIbeta. Proc. Natl. Acad. Sci. USA.

[46] Sweatt J.D. (2016). Neural plasticity and behavior—sixty years of conceptual advances. J. Neurochem..

[47] Lisman J., Yasuda R., Raghavachari S. (2012). Mechanisms of CaMKII action in long-term potentiation. Nat. Rev.. Neurosci..

[48] Okamoto K., Nagai T., Miyawaki A., Hayashi Y. (2004). Rapid and persistent modulation of actin dynamics regulates postsynaptic reorganization underlying bidirectional plasticity. Nat. Neurosci..

[49] Bosch M., Hayashi Y. (2012). Structural plasticity of dendritic spines. Curr. Opin. Neurobiol..

[50] Redondo R.L., Morris R.G. (2011). Making memories last: The synaptic tagging and capture hypothesis. Nat. Rev.. Neurosci..

[51] Bosch M., Castro J., Saneyoshi T., Matsuno H., Sur M., Hayashi Y. (2014). Structural and molecular remodeling of dendritic spine substructures during long-term potentiation. Neuron.

[52] Okamoto K., Bosch M., Hayashi Y. (2009). The roles of CaMKII and F-actin in the structural plasticity of dendritic spines: A potential molecular identity of a synaptic tag?. Physiology.

[53] Burgin K.E., Waxham M.N., Rickling S., Westgate S.A., Mobley W.C., Kelly P.T. (1990). In situ hybridization histochemistry of Ca2+/calmodulin-dependent protein kinase in developing rat brain. J. Neurosci. Off. J. Soc. Neurosci..

[54] Mayford M., Bach M.E., Huang Y.Y., Wang L., Hawkins R.D., Kandel E.R. (1996). Control of memory formation through regulated expression of a CaMKII transgene. Science.

[55] Bhattacharyya M., Stratton M.M., Going C.C., McSpadden E.D., Huang Y., Susa A.C., Elleman A., Cao Y.M., Pappireddi N., Burkhardt P. (2016). Molecular mechanism of activation-triggered subunit exchange in Ca(2+)/calmodulin-dependent protein kinase II. eLife.

[56] Stratton M., Lee I.H., Bhattacharyya M., Christensen S.M., Chao L.H., Schulman H., Groves J.T., Kuriyan J. (2014). Activation-triggered subunit exchange between CaMKII holoenzymes facilitates the spread of kinase activity. eLife.

[57] Cho M.H., Cao X., Wang D., Tsien J.Z. (2007). Dentate gyrus-specific manipulation of beta-Ca2+/calmodulin-dependent kinase II disrupts memory consolidation. Proc. Natl. Acad. Sci. USA.

[58] Incontro S., Diaz-Alonso J., Iafrati J., Vieira M., Asensio C.S., Sohal V.S., Roche K.W., Bender K.J., Nicoll R.A. (2018). The CaMKII/NMDA receptor complex controls hippocampal synaptic transmission by kinase-dependent and independent mechanisms. Nat. Commun..

[59] Hansel C., de Jeu M., Belmeguenai A., Houtman S.H., Buitendijk G.H., Andreev D., De Zeeuw C.I., Elgersma Y. (2006). alphaCaMKII Is essential for cerebellar LTD and motor learning. Neuron.

[60] Pinto T.M., Schilstra M.J., Roque A.C., Steuber V. (2020). Binding of Filamentous Actin to CaMKII as Potential Regulation Mechanism of Bidirectional Synaptic Plasticity by beta CaMKII in Cerebellar Purkinje Cells. Sci. Rep..

[61] Yin P., Xu H., Wang Q., Wang J., Yin L., Xu M., Xie Z., Liu W., Cao X. (2017). Overexpression of betaCaMKII impairs behavioral flexibility and NMDAR-dependent long-term depression in the dentate gyrus. Neuropharmacology.

[62] Frankland P.W., Bontempi B., Talton L.E., Kaczmarek L., Silva A.J. (2004). The involvement of the anterior cingulate cortex in remote contextual fear memory. Science.

[63] Akita T., Aoto K., Kato M., Shiina M., Mutoh H., Nakashima M., Kuki I., Okazaki S., Magara S., Shiihara T. (2018). De novo variants in CAMK2A and CAMK2B cause neurodevelopmental disorders. Ann. Clin. Transl. Neurol..

[64] Novak G., Seeman P., Tallerico T. (2000). Schizophrenia: Elevated mRNA for calcium-calmodulin-dependent protein kinase IIbeta in frontal cortex. Brain Res. Mol. Brain Res..

[65] Novak G., Seeman P., Tallerico T. (2006). Increased expression of calcium/calmodulin-dependent protein kinase IIbeta in frontal cortex in schizophrenia and depression. Synapse.

[66] Novak G., Fan T., O’Dowd B.F., George S.R. (2013). Postnatal maternal deprivation and pubertal stress have additive effects on dopamine D2 receptor and CaMKII beta expression in the striatum. Int. J. Dev. Neurosci. Off. J. Int. Soc. Dev. Neurosci..

[67] Greenstein R., Novak G., Seeman P. (2007). Amphetamine sensitization elevates CaMKIIbeta mRNA. Synapse.

[68] Li K., Zhou T., Liao L., Yang Z., Wong C., Henn F., Malinow R., Yates J.R., Hu H. (2013). betaCaMKII in lateral habenula mediates core symptoms of depression. Science.

[69] Song Q., Fan C., Wang P., Li Y., Yang M., Yu S.Y. (2018). Hippocampal CA1 betaCaMKII mediates neuroinflammatory responses via COX-2/PGE2 signaling pathways in depression. J. Neuroinflammation.

[70] Hoffman L., Farley M.M., Waxham M.N. (2013). Calcium-calmodulin-dependent protein kinase II isoforms differentially impact the dynamics and structure of the actin cytoskeleton. Biochemistry.

